# In-Field Detection of American Foulbrood (AFB) by Electric Nose Using Classical Classification Techniques and Sequential Neural Networks

**DOI:** 10.3390/s22031148

**Published:** 2022-02-02

**Authors:** Beata Bąk, Jarosław Szkoła, Jakub Wilk, Piotr Artiemjew, Jerzy Wilde

**Affiliations:** 1 Department of Poultry Science and Apiculture, Faculty of Animal Bioengineering, University of Warmia and Mazury in Olsztyn, Sloneczna 48, 10-957 Olsztyn, Poland; teofil.wilk@uwm.edu.pl (J.W.); jerzy.wilde@uwm.edu.pl (J.W.); 2 The Department of Computer Science, University of Rzeszow, 35-310 Rzeszow, Poland; jszkola@ur.edu.pl; 3 Faculty of Mathematics and Computer Science, University of Warmia and Mazury in Olsztyn, 10-719 Olsztyn, Poland

**Keywords:** AFB, Peanibacillus larvae, gas sensor, electronic nose, k-nearest-neighbour algorithm

## Abstract

American foulbrood is a dangerous bee disease that attacks the sealed brood. It quickly leads to the death of bee colonies. Efficient diagnosis of this disease is essential. As specific odours are produced when larvae rot, it was investigated whether an electronic nose can distinguish between colonies affected by American foulbrood and healthy ones. The experiment was conducted in an apiary with 18 bee families, 9 of which showed symptoms of the disease confirmed by laboratory diagnostics. Three units of the Beesensor V.2 device based on an array of six semiconductor TGS gas sensors, manufactured by Figaro, were tested. Each copy of the device was tested in all bee colonies: sick and healthy. The measurement session per bee colony lasted 40 min and yielded results from four 10 min measurements. One 10-min measurement consisted of a 5 min regeneration phase and a 5 min object-measurement phase. For the experiments, we used both classical classification methods such as k-nearest neighbour, Naive Bayes, Support Vector Machine, discretized logistic regression, random forests, and committee of classifiers, that is, methods based on extracted representative data fragments. We also used methods based on the entire 600 s series, in this study of sequential neural networks. We considered, in this study, six options for data preparation as part of the transformation of data series into representative results. Among others, we used single stabilised sensor readings as well as average values from stable areas. For verifying the quality of the classical classifiers, we used the 25-fold train-and-test method. The effectiveness of the tested methods reached a threshold of 75 per cent, with results stable between 65 and 70 per cent. As an element to confirm the possibility of class separation using an artificial nose, we used applied visualisations of classes. It is clear from the experiments conducted that the artificial nose tested has practical potential. Our experiments show that the approach to the problem under study by sequential network learning on a sequence of data is comparable to the best classical methods based on discrete data samples. The results of the experiment showed that the Beesensor V.2 along with properly selected classification techniques can become a tool to facilitate rapid diagnosis of American foulbrood under field conditions.

## 1. Introduction

American foulbrood is a dangerous disease of the honeybee caused by the bacterium *Paenibacillus larvae larvae* (white). This bacterium produces highly resistant spores [1] and can therefore survive for decades [2]. The spores can be carried by bees with food and by the beekeeper on beekeeping equipment. In this way, the disease spreads rapidly first in the apiary from colony to colony and then from apiary to apiary.

The disease is widespread worldwide [3] and has been recorded in apiaries on five continents [4]. Monitoring studies of honey contamination with *P.l. larvae* spores conducted in Poland [5] have shown that there are regions where the risk of American foulbrood symptoms is high. An example is the Warmia and Mazury Province, where the presence of sporocarps was confirmed in almost 50% of the examined apiaries, 20% of which showed a high degree of contamination [5]. The results of this study translate into the current epizootic situation in the area (results of observations during field work by a co-author of the project, a veterinary surgeon specialising in bee diseases).

**Figure 1:**
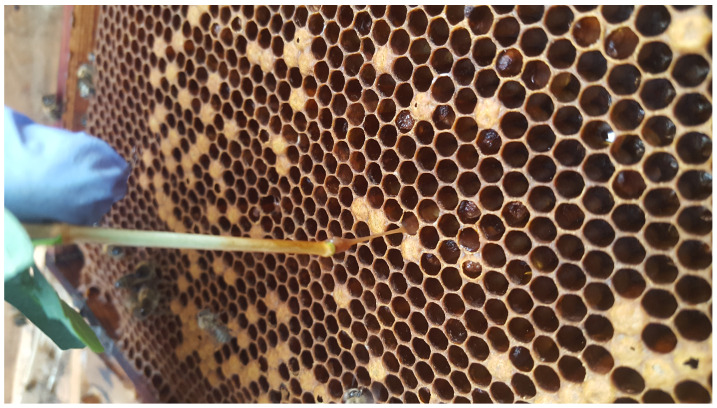
The bee comb with diseased brood for American foulbrood. Visible dragging rotten larvae, which emit a specific odour.

American foulbrood manifests itself in the dying and rotting of bee brood underneath the sealing caps (Figure 1. Consequently, this disease leads to the weakening of a strong colony and its death [6].

Beekeepers have difficulty perceiving and recognising the early symptoms of American foulbrood. As a result, they find that something bad is happening to their bee colonies when it is too late for rescue. Confusing symptoms of American foulbrood with symptoms of other diseases of sealed brood is also a problem. To confirm the presence of the disease in the apiary, laboratory diagnostics are necessary. This involves taking bee and brood samples for testing, protecting them properly, sending them to a laboratory, and waiting a long time for the results. Such tests are often expensive, especially when the apiary is large, and the number of samples sent for laboratory tests must also be significant. Other tools are therefore being sought for rapid and cheap diagnosis of American foulbrood, which will alert the beekeeper to the problem at an early stage of the disease under field conditions.

Our study took advantage of the fact that rotting brood produces a mixture of valerian, isocaproic, and caproic acids [7]. This causes a specific odour to be emitted by a colony suffering from American foulbrood, which can even be detected by the human nose. It therefore made sense to use an electronic nose to detect American foulbrood. The effectiveness of electronic nose detection has been scientifically proven many times. These devices have found applications in the diagnosis of diseases in plants [8], animals [9], and humans [10]. In the case of bee diseases, an array of six solid-state gas sensors can distinguish bee colonies heavily infected with the *Varroa destructor* parasite from healthy colonies [11] and can diagnose Varroa by examining brood samples infected with this mite [12]. The effectiveness in detecting American foulbrood in bee colonies by the multi-sensor device in preliminary studies was confirmed by a team of scientists from Australia [13].

The study tested the effectiveness of the Beesensor V.2 multi-sensor recorder in detecting American foulbrood in bee colonies and thus to answer the question of whether this electronic nose could become an effective tool in diagnosing American foulbrood.

## 2. Materials and Methods

### 2.1. Multi-Sensor Recorder Beesensor V.2

**Figure 2:**
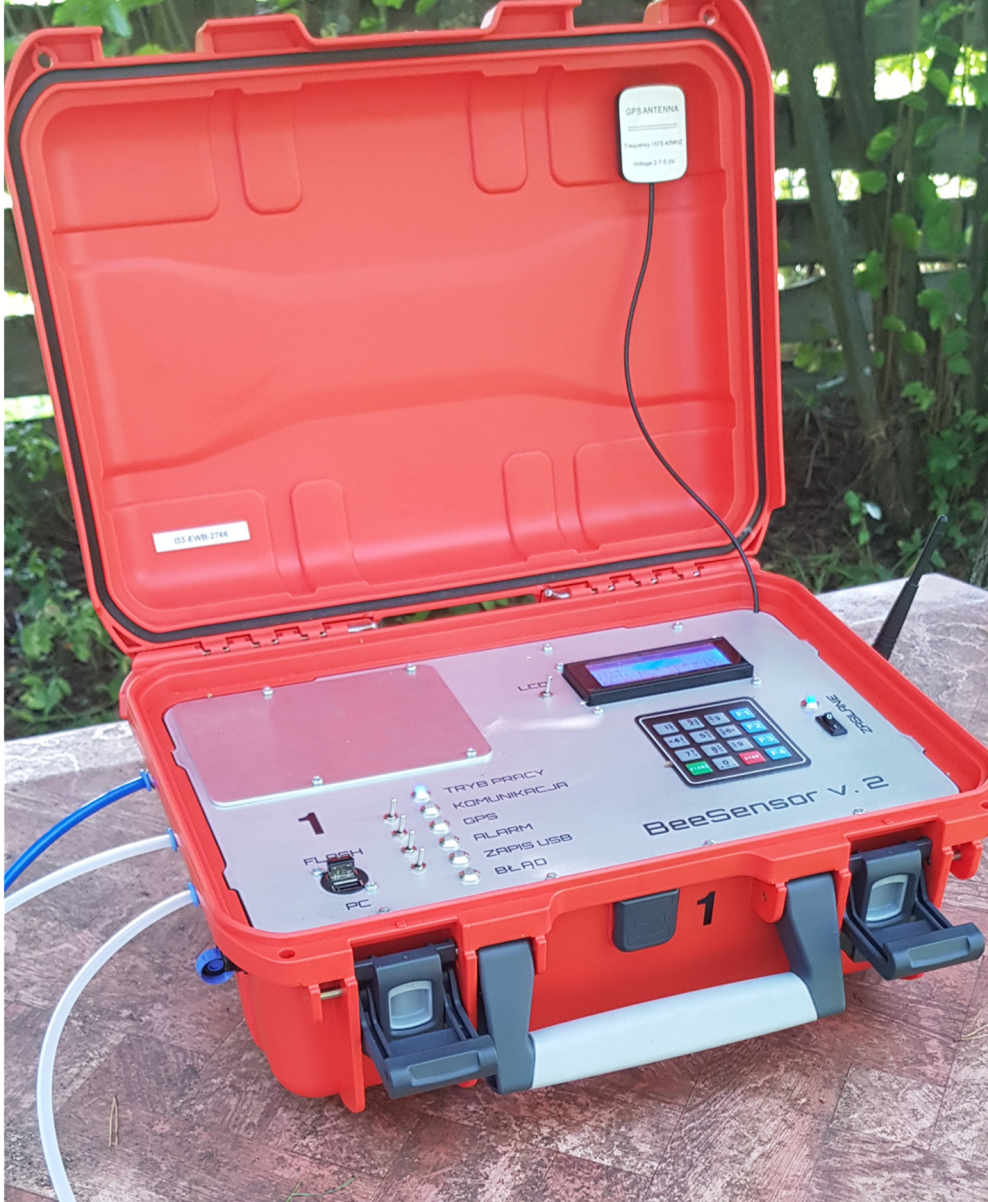
Multi-Sensor Recorder Beesensor V.2.

In the experiment, three units of Beesensor V.2 (*Beecom* 1, *Beecom* 2, and *Beecom* 3) constructed at the Wrocław University of Technology in the Laboratory of Sensor Techniques and Indoor Air Quality Research were tested (Figure 2). Beesensor V.2 is an advanced multi-sensor signal recorder based on semiconductor gas sensors TGS823, TGS826, TGS832, TGS2600, TGS2602, and TGS2603 from FIGARO. The sensing layer of the Taguchi Gas Sensors (TGS) used in the experiment was SnO_2_. This MOS is more sensitive than others and can operate already at 300 degrees Celsius. The sensitivity and selectivity of TGS sensors is achieved by enriching SnO_2_ with various chemical elements, including noble metals (Pt, Pd, and Ru) or rare earth metals (Y). However, the exact composition and proportions of added elements of individual TGS sensor models are a trade secret of the company. Each sensor reacts to the presence of different substances (Table 1).

The device has two inputs (regeneration path and measuring path) and one gas outlet provided with quick plug connectors. Beesensor V.2 is built up of several modules. The pneumatic module is responsible for gas sampling and sample preparation. The essence of this module is a gas pump, which supplies gas samples to the sensor block by using Teflon tubes. A carbon filter is placed in the reference path and a dust filter composed of a mixture of cellulose ester (MCE 13 mm, 0.45 microns) in the measurement path.

The user interface is the communication module, which is made up of the following elements:

- Main switch,
- LCD display,
- Alphanumeric keypad,
- Four rocker switches controlling GPS, USB, GSM, and sound
- Optical controls (LEDs),
- Loudspeaker,
- USB sockets.

Furthermore, the device includes a microcomputer, microcontroller, GSM and GPS communication modules, and an internal clock. Beesensor V.2 is equipped with a stand-alone power module consisting of a battery with a capacity of 22 Ah and a voltage of 12 V, so it can be used independently of the access to main power.

Beesensor V.2 has software modules for data acquisition, processing, and transmission. Furthermore, this multi-sensor recorder is fully controllable and programmable. It enables identification of the class of tested samples thanks to the possibility of introducing a classifier, in which the method of construction of the feature vector is the selection of a moment from the time series containing measurement data for each sensor independently. The classifier can be trained by pointing to files containing reference data for a given class from the previously performed measurements under controlled conditions. Based on the indicated feature vector and the previously built set of benchmarks, the classifier calculates the class and reports it to the user.

### 2.2. Experimental Scheme

The study was conducted in September 2020 in an apiary located in the Warmińsko–Mazurskie (Poland) province. This apiary consisted of 18 bee colonies (*Apis mellifera carnica*), of which 9 colonies showed symptoms of American foulbrood. Official laboratory tests on samples of the brood taken from the affected colonies confirmed infection with *P.l. larvae*. Bee colonies were housed in Warsaw beehives. The average strength of sick colonies was 7.3 combs, while healthy colonies were occupying an average of 10.3 combs. The bee colonies were classified according to their health status. Thus, two classes were distinguished:

- Class 0—colonies suffering from American foulbrood (visible clinical symptoms; the disease was confirmed by laboratory diagnostics)—9 objects,
- Class 1—healthy colonies—9 objects.

**Figure 3:**
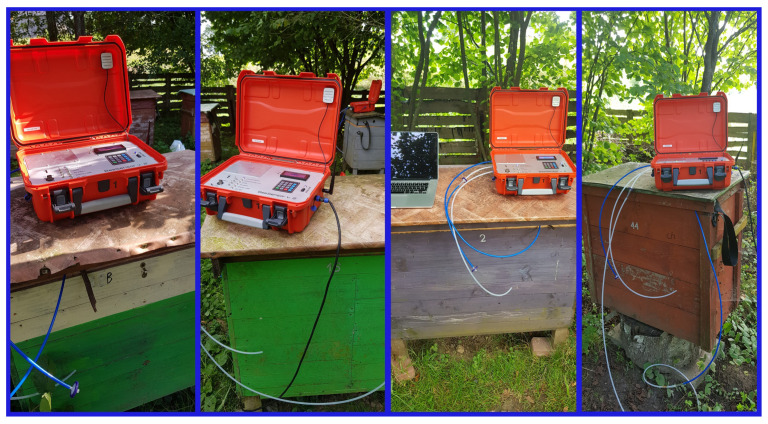
Multi-sensor recorder Beesensor V.2, unit BEECOM 1, during the measurement session of the different bee colonies.

In the experiment, three twin units of Beesensor V.2 called: *Beecom* 1, *Beecom* 2, and *Beecom* 3 were used. Each unit was warmed up for at least 12 h before measurements were taken. Eighteen measurement sessions were performed. Each unit of the device measured a different colony in a single measurement session, and in subsequent sessions, it alternately measured a healthy and a sick colony (Figure 3). The probe tip was placed in the middle of the bee nest, between two brood combs. The object of measurement was a gas sample from this inter-comb space.

The research lasted 3 days. The main assumption of the measurements was to examine each bee colony by each device in similar conditions of the same apiary, hive type, and management method. Unfortunately, in the fifth session, the *BEECOM* 3 device crashed, which caused it to examine only 17 bee colonies: 9 sick and 8 healthy (Table 2).

The single measurement procedure was already implemented in each device. The parameters of the device for performed measurements are shown in the Table 3.

A single measurement session per bee colony lasted 40 min and consisted of four individual measurements. For simplicity in data handling, the measurements were named “beex.y” where “bee” is the abbreviation for *BEECOM*; “x” is the number of the next Beesensor V.2; and “y” is the next measurement in the measurement session, whereby the x value x∈{1,2,3} and the value y∈{0,1,2,3}. Each measurement lasted 10 min, of which 5 min were the regeneration (cleaning) phase, and 5 min were the object-measurement (scent saturation) phase (Table 4). During the regeneration phase, the devices sampled pure air outside the hive, and during the measurement phase, gas samples were collected from the centre of the bee nest, from between two brood patches. Measurements covering the first 20 min of the measurement session, where y = 0 and y = 1, were not taken for analysis at all, as they gave unstable readings.

### 2.3. Data Processing

**Figure 4:**
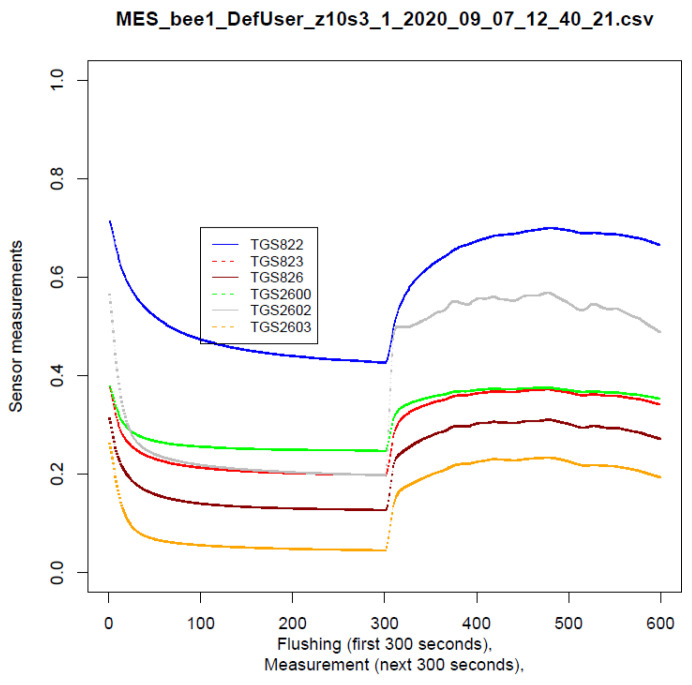
An example of a 600 s gas sensor reading, representing a single measurement. It shows areas where the readings stabilise. The stable areas provide the basis for selecting values for the classification process.

One reading of the sensor is understood as taking the average volt recorded by the sensor during 1 s. The result of measurement session one for the bee colony was to obtain four raw measurement files. One file with a given device consists of 300 s of cleaning and 300 s of scent saturation. Both phases have their stabilisation intervals; in the cleaning phase, the minimum value was reached, and in the saturation phase, the maximum value was reached. An example of a reading in the cleaning and saturation phases can be seen in Figure 4.

## 3. The Experimental Part Design

In the field tests we had access to nine bee colonies of each class. The differences in the size of sites in the classes for devices and reading numbers were due to some difficulties encountered during the tests. In class 0, we considered colonies with numbers: 1, 2, 3, 6, 13A, 13B, 19, 20, and 44. In class 1, we considered colonies with numbers: 5, 9, 10, 11, 11B, 12, 14, 15, and 16. The colony numbers we use for each test are in Table 5.

**Figure 5:**
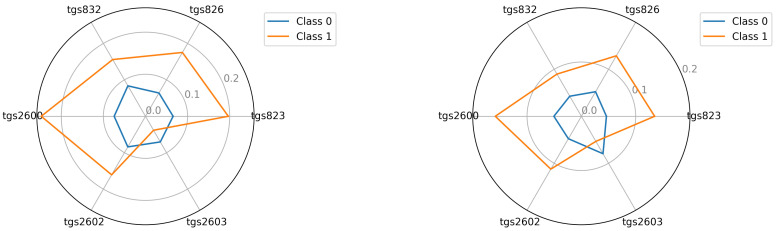
Visualisation for device bee1: Squared the average reading in classes 0 and 1; bee1.2 and bee1.3 (**left**) and (**right**), respectively.

**Figure 6:**
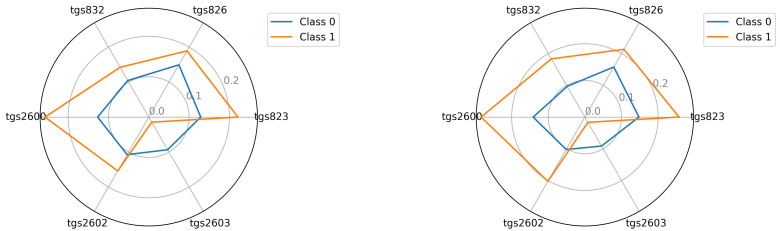
Visualisation for device bee2: Squared the average reading in classes 0 and 1; bee2.2 and bee2.3 (**left**) and (**right**), respectively.

**Figure 7:**
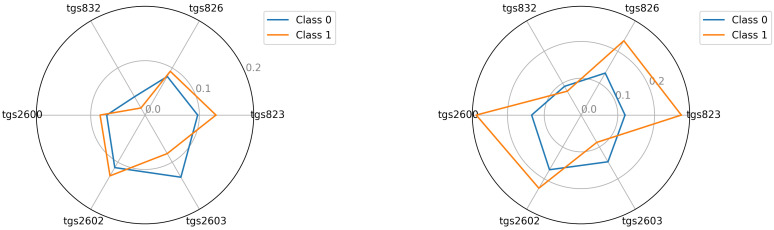
Visualisation for device bee3: Squared the average reading in classes 0 and 1; bee3.2 and bee3.3 (**left**) and (**right**), respectively.

The starting point for investigating classification possibilities is to visualise the average readings of the individual TGS sensors—see Figure 5, Figure 6 and Figure 7. We visualised the data before and after the baseline correction. We used the Multiple Train and Test method (Monte Carlo Cross Validation technique [14,15]) to verify the quality of the classification models. We performed 25 tests where each split is applied to all classifiers simultaneously. To assess the quality of the results, we used the balanced parameter accuracy [16], which is the average classification accuracy of all classes. As an auxiliary parameter, we used the true-positive rate (TPR), i.e., the percentage hit in the class.

In the experimental part, we test the effectiveness of classical classification techniques (applied on extracted data with a selected strategy) and a technique based on the analysis of data sequences using neural networks. Let us start with our approach in the context of the use of classical techniques.

### 3.1. Classical Classifiers—Using an Extracted Representation of the Data

For an algorithmic basis, we used the classifier: 1nn (see [17]), nb ([18], Devroye et al. [19], and Duda et al. [20]), Support Vector Machine (svm) with fixed kernels, svm_linear [21], svm_radial [21], svm_polynomial [21], svm_sigmoid [21], Generalized Linear Regression (lg) [22], Random Forests (rf) [23] and the Committee of Classifiers (com3) [24]. We also used a classification committee as a tuning element.

**Figure 8:**
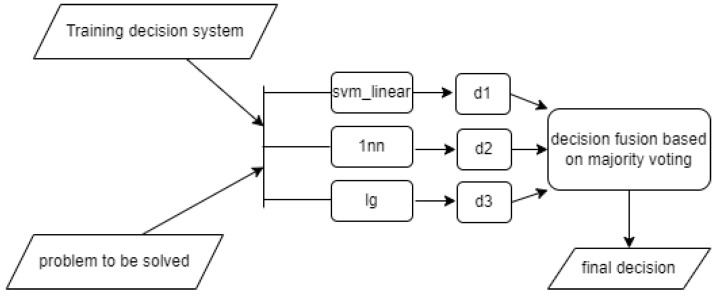
Diagram showing the operation of the com3 method.

All classification methods used in the study are sourced from the R language [25] packages. They were used with default settings. The classification committee—i.e., the com3 method—was designed based on the 1nn, svm_linear, and lg methods—see the scheme in Figure 8.

**Figure 9:**
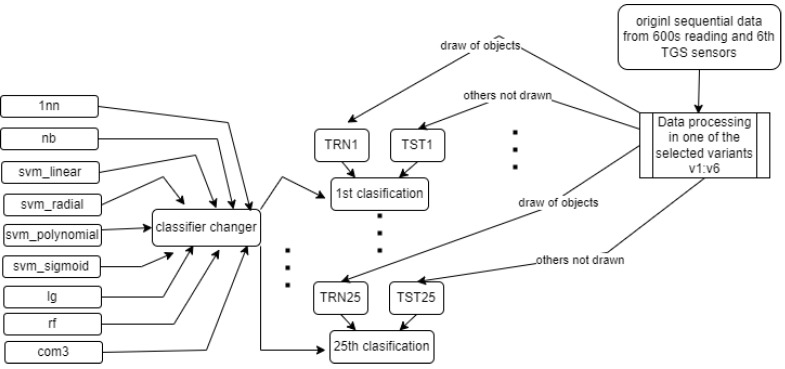
The general scheme of our experiments.

The general scheme of the research carried out is shown in Figure 9.

#### 3.1.1. Variants of Data Preprocessing

Assuming *j* is the bee colony number, *i* the TGS sensor number, i∈{823,826,832,2600,2602,2603}, and s the measurement second number, we defined the following variants of the test methodology.

**VARIANT1:**

In this variant, we applied the following transformation.

$$TGSnew_{i}\left(object_{j}\right)=\frac{\sum_{s=521}^{580}TGS_{i}^{s}\left(object_{j}\right)}{60}$$

**VARIANT2:**

In this variant, we applied the following transformation, which we call the **baseline correction**.

$$TGSnew_{i}\left(object_{j}\right)=\frac{\sum_{s=521}^{580}TGS_{i}^{s}\left(object_{j}\right)}{60}-\frac{\sum_{s=281}^{290}TGS_{i}^{s}\left(object_{j}\right)}{10}$$

**VARIANT3:**

This time we used maximum readings in the scent saturation phase.

$$TGSnew_{i}\left(object_{j}\right)=max_{s=301}^{599}TGS_{i}^{s}\left(object_{j}\right)$$

**VARIANT4:**

In the fourth variant, we had a baseline correction based on subtracting from the maximum reading of the saturation phase the minimum reading of the cleaning phase.

$$TGSnew_{i}\left(object_{j}\right)=max_{s=301}^{599}TGS_{i}^{s}\left(object_{j}\right)-min_{s=1}^{299}TGS_{i}^{s}\left(object_{j}\right)$$

**VARIANT5:**

In this variant, we combined the attributes from variants 1 and 2. The result was a system with twelve conditional attributes.

**VARIANT6:**

In the final variant, we combined the conditional attributes from variants 3 and 4. The result was an analogous extended system as in variant 5.

#### 3.1.2. Results of the Experiments for Classical Techniques

##### Analysis of the Results for the 25 Times Train and Test Method

First, we used six baseline research methodologies—see Section 3.1.1—and 9 reference classification techniques.

The testing methodology we chose to evaluate the models with was to split the entire sample set 25 times into training and test systems, using a split ratio of 0.6. This solution was chosen because the tested decision system contains less than 20 test samples and because classical cross-validation cannot be applied. During testing, the selected classifiers were applied to the same data splits, and the results were averaged. We used the balanced accuracy as a parameter to measure the quality of classification and the sensitivity and significance (specificity) of the tests, i.e., the percentage effectiveness in indicating the class of diseased samples and the class of healthy samples. An example of the detailed result for the best variant can be seen in Table 6, Table 7, Table 8, Table 9, Table 10 and Table 11. It is worth noting that the third and fourth measurements from devices *BEECOM* 1, *BEECOM* 2, and *BEECOM* 3, marked as bee1.2, bee1.3, bee2.2, bee2.3, bee3.2, and bee3.3 were selected as reference measurements because they showed stabilised readings compared to the first and second measurements. Let us move on to a summary of the experimental part.

##### Summary of Results

In Table 12, Table 13 and Table 14, we have a summary of the results for the tested classification techniques and the v1:v6 test methodologies. We have results for three classification quality intervals (accuracy-balanced) 0.6, 0.65, and 0.7. The best result encountered was 0.748 for the svm_linear method and the v4 variant. Variant v4 won slightly, but the second best was variant v2. The best classification method was found to be the svm_linear technique, which performed stably in all tested variants. The second best was com3 (i.e., a committee of three classifiers 1nn, svm_linear and lg).

### 3.2. v4: Results with BASELINE CORRECTION Max Measure between 301:599 Minus Min Measure between 1:299

Seeing the experimental results on the tested data, given the fact of having few samples, we decided to perform an additional visualisation of the mean class values in the best, variant 4, so far.

### 3.3. Classification Using a Sequential Neural Network—Using the Full Sequence of Data

#### 3.3.1. Description of the Input Data

The input data are a set of samples containing multivariate values of variables over time; for each sample, we assigned one of two decisions that classify this sample appropriately. Each sample contains 600 s of time-varying data and contains six attributes—values from the following sensors: TGS823, TGS826, TGS832, TGS2600, TGS2602, and TGS2603.

**Figure 10:**
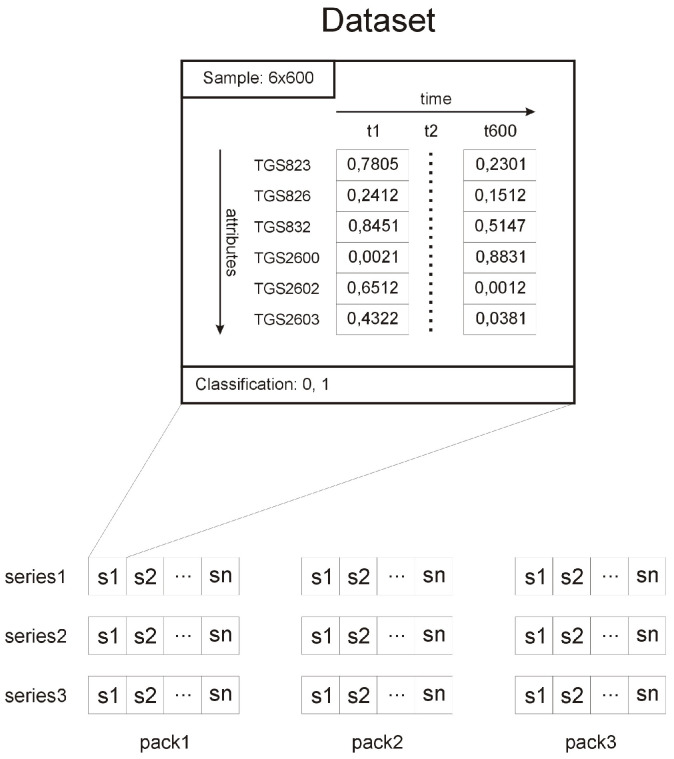
Data structure.

**Figure 11:**
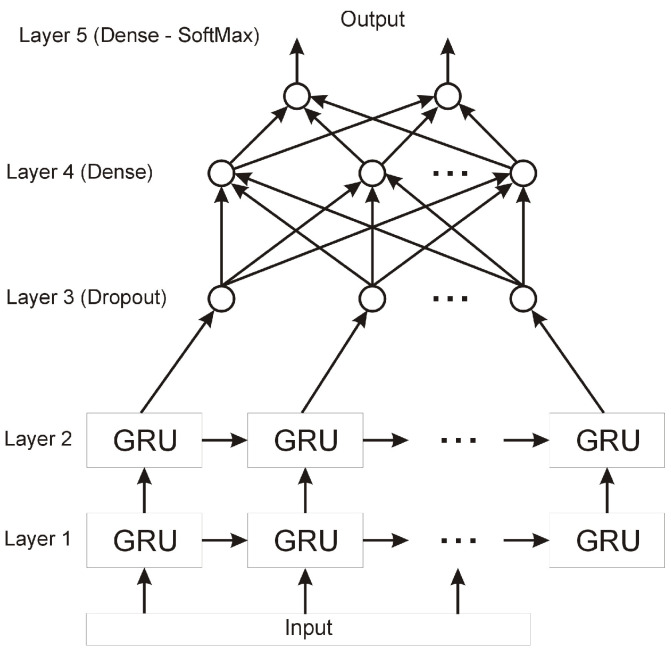
Neural network model.

The input data are organised as follows: we had three sets of measurement data: bee1, bee2, and bee3 coming from different units of the device: BEECOM1, BEECOM2, and BEECOM3, respectively. Each data set contains three consecutive data subsets from successive measurements that were collected in short intervals; the whole process is related to the specificity of the measurement data. Due to the need to stabilise the level of odours, and the need to obtain as far as possible undisturbed data with initial conditions, the first data subgroup called “pack1” was rejected; the model training and prediction process used data from three units of device (bee1, bee2, and bee3) and from two measurements (measurement2 and measurement3). For each 600 s data sequence, we had one decision assigned, which clearly determined whether the sample belonged to healthy bee colony or whether we were dealing with a disease entity. The decision data came from experts. To create a data model that is useful in the diagnosis or prognosis of American foulbrood, a sequential neural network was proposed, composed of many layers of appropriately selected neurones and processing units. In the case of neural networks that are in favour of analysing data composed of long data sequences, architectures are used that show high resistance to fading or exploding gradient problems. Examples of such networks are architectures that use LSTM or GRU cells. The presented architecture uses Gated Recurrent Unit (GRU) units, due to their simpler structure compared to LSTM units, their having a similar performance, and their ability to learn long data sequences. The structure of the data used and the visualisation of our model can be seen in Figure 10 and Figure 11.

The proposed neural network architecture includes five layers. The first two layers are GRUs 150 in the first layer and 50 in the second layer, respectively. Then, there are dropout units with a coefficient of 0.5, whose task is to ensure appropriate network regularisation. This process affects the quality of learning, which is to minimise the possibility of overfitting the network. The next layer is a layer of 10 fully connected units and the last decision layer containing two output neurones. Because the network is to be used for the classification of the input samples, the softmax activation function was used in the last layer, which provides an indication of the decision class with a certain probability.

#### 3.3.2. Results for Sequential Network

The input data were divided into training and test data in the proportions of 90% and 10%, respectively.

The training data included 600 s of six-element vectors in one training iteration (batch_size = 600, features = 6), and the number of training iterations was set to 200 (epochs). Then, after training the network, the model was validated using test data. The network has two exits, and each of them determines the group’s affiliation to a given class (healthy or sick). For training the network, 117 records were used, while 11 were for testing. In total, 421,200 values were used for training the network and 39,600 values for testing. The model for test samples showed an accuracy of 72.72%—for details see Table 15.

### 3.4. The Visualisation of the Tested Classes Based on the Average Intensity of TGS Sensor Readings for Data Prepared According to Variant 4

**Figure 12:**
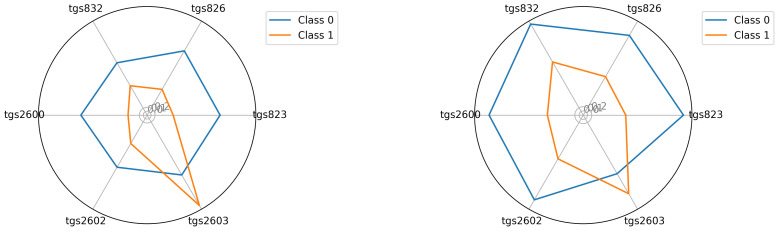
Visualisation for device bee1: The average readings were logarithmised and squared in classes 0 and 1; bee1.2 and bee1.3 (**left**) and (**right**), respectively.

**Figure 13:**
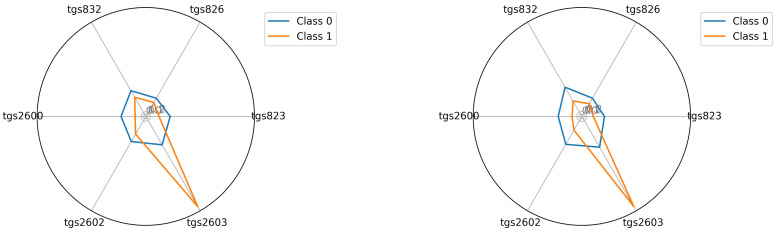
Visualisation for device bee2: The average readings were logarithmised and squared in classes 0 and 1; bee2.2 and bee2.3 (**left**) and (**right**), respectively.

**Figure 14:**
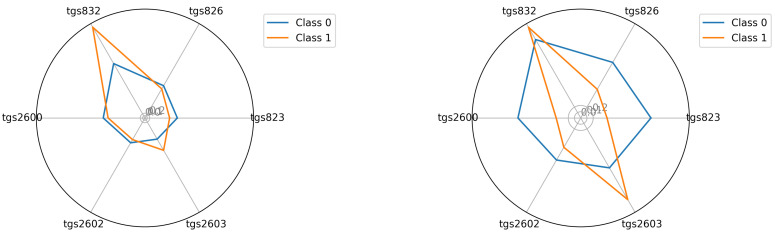
Visualisation for device bee3: The average readings were logarithmised and squared in classes 0 and 1; bee3.2 and bee3.3 (**left**) and (**right**), respectively.

In this section, we illustrate the visual differences between the 0 and 1 classes. The visualisations presented support the claim that the artificial nose can distinguish between sick and healthy bee colonies. Visualisation is an important support because the number of samples we had did not allow to statistically verify this thesis with certainty. We considered two options for visualisation. In the first option—see Equation (2)—we presented the squared mean sensor readings of the decision classes. See Figure 5, Figure 6 and Figure 7. In the second option—see Equation (3)—we used logarithmic averages and squared them—see Figure 12, Figure 13 and Figure 14.

(1) $$average\left(TGS_{i}^{class_{j}}\right)=\frac{\sum_{k=0}^{|class_{j}|}TGS_{j}\left(ob_{l}\right)}{|class_{j}|},\;where\;ob_{l}\in class_{j}$$

(2) $$In\;the\;first\;option,\;we\;used:\;{\left(average\left(TGS_{i}^{class_{j}}\right)\right)}^{2}$$

(3) $$In\;the\;second\;option,\;we\;used:\;{\left(log\left(average\left(TGS_{i}^{class_{j}}\right)\right)\right)}^{2}$$

## 4. Discussion

Electric noses based on metal oxidesemiconductors (MOS) have been used with good detections for many human diseases such as the following: urinary tractinfections [26], cancer [27], diabetes [28], and bowel diseases [29,30]. They also work well in detecting animal diseases. Fend et al. 2005 [31] used successfully an electronic nose to diagnose *Mycobacterium bovis* infection in badgers and cattle. Devices based on semiconductor sensors operation have also been successful in veterinary diagnostics in cases: Acute liver failure of rats [32], *Cutaneous myiasis* of sheep [33], and white-nose syndrome of cave-dwelling bats [9].

Our team focused on the ability to detect the most dangerous honeybee diseases such as varrosis and American foulbrood. The Figaro 6-sensor TGS device we used has so far given satisfactory results in Varroa diagnostics, both in the laboratory (ref. [12] and in the field conditions [11]. We also successfully detected colonies on MYPGP media *P.l. larvae* [34]. In this article, we presented the results of using a BeesensorV.2 device based on an array of the same six sensors to detect American foulbrood under field conditions in live bee colonies. The diagnostic studies of American foulbrood under field conditions have not been conducted by anyone until now. This is completely innovative research. The team of scientists Moran i in. (2019) [13] did, by gas chromatography mass spectrometry (GC-MS), preliminary identification of volatile compounds (VCs). These were to become AFB volatile biomarkers and to be used in the future as indicators of diagnostic electronic nose. However, further results of the study were not presented. It is also not known on which sensors the device was to be based.

The analysis of the graphs showing the time-course readings of the individual results has shown that the readings from the first two measurements are unstable in many cases. In particular, the first measurement marked as 0 stands out from the next three measurements. This is because at the beginning of sampling, the bee colony gas was beginning to saturate with odour. Only the third and fourth measurements were stable. Thus, we can conclude that to diagnose American foulbrood, BeesensorV.2 cannot work on a single colony for less than half an hour. It must be remembered that these are field conditions. The situation is different in the case of laboratory tests, where the array of the same sensors already gave stable readings in the first and only measurement (10 min of measurement session) [12,34,35].

The nine classification techniques were selected for data analysis. The best method was svm_linear. It gave the highest efficiency of class distinction for samples of *BEECOM* 1 (75%) (Table 6) and *BEECOM* 2 (74%) (Table 8). Support vector machines (SVM) is an efficient classifier, which is often used by other data scientists [36]. Good classification results were obtained with this classifier in earlier studies on Varroa diagnosis [11]. For *BEECOM* 3, the best results were obtained with the lg method. A classification efficiency of 71% was obtained (Table 11. Considering all the analyses for the three units and all the third and fourth measurements, a good classification tool becomes the classification committee based on the three techniques of 1nn, lg, and svm_linear.

The performance of the nine classifiers tested was subjected to both raw data and data with baseline correction performed using different techniques. These measures are described as variants 1–6 Section 3.1.1 The best results were obtained for variants 4 and 2, i.e., with a baseline correction. Many researchers recommend baseline correction for sensor readings that may be affected by environmental factors [37,38,39,40]. The bee colony is an active and rich-in-volatile-compounds (VCs) organism. Its smell can particularly change in late spring and summer when the bees are working intensively. We deliberately conducted our experiment in autumn months and on cold days (13 °C). Bee activity was low then, and there were no additional scent factors both inside the bee nest (e.g., influx of fresh nectar and swarming) and outside the hive (flowering of intensely scented plants) that could significantly interfere with the sensor readings. Additionally, the timing of the experiment helped reduce to zero the possibility of triggering a robbery in the apiary, which could contribute to the transmission of American foulbrood from sick to healthy colonies.

When testing different classification methods, we could not combine the results of three different units of the same device, so we had to treat the data from each unit individually. This is due to a phenomenon called sensors drift [41]. It is a certain imperfection of MOS sensors, where individual copies of the same sensor, used in the same conditions, do not give identical readings. The visualisation of the squared average reading according to variant 4 proved that the array image of the sensor readings obtained from the three copies of BEECOM1, BEECOM2, and BEECOM3 are different Section 3.4. Therefore, each piece of semiconductor sensor-based equipment requires an individual approach to calibration, e.g., by using a special algorithm [42]. We proceeded differently in the case of the sequential neural network. Here, the results from three pieces of equipment were finally treated collectively. However, in the learning phase, the classified objects were separated. The sequential neural network gave us satisfactory results of classification on the level of 73%. This result was fully consistent with the average classification result obtained by the best classifiers for the three BeesensorV.2 units. We must remember that in a bee colony, different volatile compounds (VCs) are released by both elements of the hive environment (honey bee, pollen, wax, and propolis) and by living organisms: bees and brood. Therefore, we are dealing with a gas that is a mixture of several dozen substances, and we conducted research on various bee diseases in field conditions. Thus, it was found that the device could not be based on a single sensor. A matrix of six sensors was created, each of which reacts to different substances. We analysed the matrix image of the complicated gas coming from the tested object. It was therefore concluded that the best method of device calibration would be the use of learning algorithms. The best classifiers for a particular device will be trainable.

In conclusion, Beesensor V.2 along with appropriate classification techniques showed great potential as a tool to distinguish bee colonies affected by American foulbrood from healthy ones. Thus, this electronic nose can become an effective tool for diagnosing this extremely dangerous disease in the apiary. Rapid detection of infection *P.l. larvae* in a single bee colony will allow the beekeeper to react immediately. In this way, it will prevent further spread of the disease to other bee colonies and neighbouring apiaries. This will save the apiary from extinction.

## 5. Conclusions

1. Beesensor V.2 distinguishes between bee colonies infected with American foulbrood and healthy bee colonies at a level of 73%.
2. During the field tests, the third and fourth measurements out of four measurements, which is the result of the measurement procedure implemented in the device, proved to be the most stable regardless of the device used.
3. As the third measurement was already stable, the time of measurement of a single bee colony with Beesensor V.2 could be shortened to 30 min.
4. A baseline correction was required to obtain optimal classification results. Both winning variants v4 and v2 use it.
5. The svm classifier with a linear kernel (svm_linear method) proved to be the best tool for classification (among classical methods) in the context studied. The second most stable method was the classification committee based on the three techniques: 1nn, lg, and svm_linear.
6. The results of data analysis using sequential neural networks on the entire data series were found to be comparable with the results for the best classical methods, which are based on a baseline correction and an extracted discrete data sample.

## Figures and Tables

**Table 1:** The characteristics of semiconductor gas sensors, which were used in the multi-sensor array ([12], https://www.figaro.co.jp/en (accessed on 4 December 2021)).

Sensor	Substances Detected	Detection Range
*TGS*823	Organicsolventvapors	50∼5000ppm,ethanol,n-hexane,benzene,acetone
TGS826	Ammonia	30∼300ppm,ethanol,ammonia,isobutane
TGS832	Chlorofluorocarbons	100∼3000ppm,R-407c,R-134a,R-410a,R-404a,R-22
TGS2600	Gaseousaircontaminants	1∼100ppm
TGS2602	VOCsandodorousgases	1∼30ppm,ethanol,ammonia,toluene
TGS2603	Amine-seriesandsulfurousodorgases	1–30ppm,ethanol,0.1–3ppmtrimethylamine
		0.3–2ppmmethylmercaptan

**Table 2:** The schedule of measurements taken, stating that each device shall test alternately sick and healthy bee colonies, and each device shall test all bee colonies participating in the experiment.

Session	*BEECOM* 1		*BEECOM* 2		*BEECOM* 3		Data
	No. Bee Colony	Health Status	No. Bee Colony	Health Status	No. Bee Colony	Health Status	
1	44	sick	10	healthy	1	sick	07.09.2020
2	10	healthy	44	sick	5	healthy	07.09.2020
3	1	sick	5	healthy	44	sick	07.09.2020
4	5	healthy	1	sick	10	healthy	07.09.2020
5	2	sick	11	healthy	-		07.09.2020
6	11	healthy	2	sick	3	sick	07.09.2020
7	3	sick	12	healthy	11	healthy	07.09.2020
8	12	healthy	3	sick	2	sick	8.09.2020
9	6	sick	14	healthy	12	healthy	8.09.2020
10	14	healthy	6	sick	20	sick	8.09.2020
11	20	sick	15	healthy	14	healthy	8.09.2020
12	15	healthy	20	sick	6	sick	9.09.2020
13	19	sick	16	healthy	15	healthy	9.09.2020
14	16	healthy	19	sick	13A	sick	9.09.2020
15	13A	sick	9	healthy	16	healthy	9.09.2020
16	9	healthy	13A	sick	19	sick	9.09.2020
17	13B	sick	11B	healthy	9	healthy	9.09.2020
18	11B	healthy	13B	sick	13B	sick	9.09.2020

**Table 3:** The measurement parameters.

Pump power	30%
Temperature in the measuring chamber	39 °C
Humidity in the measuring chamber	25% (21–29%)
Temperature in the central part of the bee colony nest	35 °C
Temperature outside the hive	13 °C (11°–15°)

**Table 4:** The scheme of the measurement session including the duration of each phase and the names of the obtained measurements. Legend: reg.—regeneration phase; meas.—measurement phase.

Device	Measurement Session (t = 2400 s)							
	Measurement 0 (t = 600 s)		Measurement 1 (t = 600 s)		Measurement 2 (t = 600 s)		Measurement 3 (t = 600 s)	
	reg. (t = 300 s)	meas. (t = 300 s)	reg. (t = 300 s)	meas. (t = 300 s)	reg. (t = 300 s)	meas. (t = 300 s)	reg. (t = 300 s)	meas. (t = 300 s)
*BEECOM* 1	bee1.0		bee1.1		bee1.2		bee1.3	
BEECOM2	bee2.0		bee2.1		bee2.2		bee2.3	
BEECOM3	bee3.0		bee3.1		bee3.2		bee3.3	

**Table 5:** The number of bee colonies correctly examined during each test. Only this number of colonies was used in the tests. $no.of.col.in.class_{i}$ = number of colonies available in $class_{i}$, i∈{0,1}.

Device	Measurement	$\mathrm{no}.\mathrm{of}.\mathrm{col}.\mathrm{in}.{\mathrm{class}}_{0}$	$\mathrm{no}.\mathrm{of}.\mathrm{col}.\mathrm{in}.{\mathrm{class}}_{1}$
*BEECOM 1*	bee1.1	7	9
	bee1.2	7	9
	bee1.3	7	9
BEECOM2	bee2.1	9	9
	bee2.2	9	9
	bee2.3	9	9
BEECOM3	bee3.1	9	9
	bee3.2	9	9
	bee3.3	9	8

**Table 6:** Summary of results for **data.bee1.2** with baseline correction.

Method	$b_{\mathrm{acc}}$	${\mathrm{acc}}_{0}$	${\mathrm{acc}}_{1}$
1nn	**0.692**	0.653	0.73
nb	0.575	0.6	0.55
svm_linear	**0.748**	0.667	0.83
svm_radial	**0.677**	0.493	0.86
svm_polynomial	0.587	0.213	0.96
svm_sigmoid	**0.683**	0.547	0.82
lg	0.565	0.6	0.53
rf	**0.702**	0.613	0.79
com3	**0.73**	0.68	0.78

**Table 7:** Summary of results for **data.bee1.3** with baseline correction.

Method	$b_{\mathrm{acc}}$	${\mathrm{acc}}_{0}$	${\mathrm{acc}}_{1}$
1nn	0.543	0.547	0.54
nb	0.453	0.387	0.52
svm_linear	**0.688**	0.547	0.83
svm_radial	0.485	0.12	0.85
svm_polynomial	0.512	0.173	0.85
svm_sigmoid	0.452	0.133	0.77
dt	0.5	0	1
lg	**0.713**	0.707	0.72
rf	0.502	0.453	0.55
com3	**0.675**	0.6	0.75

**Table 8:** Summary of results for **data.bee2.2** with baseline correction.

Method	$b_{\mathrm{acc}}$	${\mathrm{acc}}_{0}$	${\mathrm{acc}}_{1}$
1nn	**0.6**	0.63	0.57
nb	**0.625**	0.68	0.57
svm_linear	**0.735**	0.67	0.8
svm_radial	0.595	0.7	0.49
svm_polynomial	0.535	0.46	0.61
svm_sigmoid	0.585	0.58	0.59
lg	**0.6**	0.61	0.59
rf	0.53	0.54	0.52
com3	**0.655**	0.63	0.68

**Table 9:** Summary of results for **data.bee2.3** with baseline correction.

Method	$b_{\mathrm{acc}}$	${\mathrm{acc}}_{0}$	${\mathrm{acc}}_{1}$
1nn	0.59	0.58	0.6
nb	**0.605**	0.71	0.5
svm_linear	**0.685**	0.6	0.77
svm_radial	**0.6**	0.57	0.63
svm_polynomial	**0.61**	0.34	0.88
svm_sigmoid	0.55	0.5	0.6
lg	0.595	0.57	0.62
rf	0.53	0.54	0.52
com3	**0.66**	0.59	0.73

**Table 10:** Summary of results for **data.bee3.2** with baseline correction.

Method	$b_{\mathrm{acc}}$	${\mathrm{acc}}_{0}$	${\mathrm{acc}}_{1}$
1nn	**0.645**	0.74	0.55
nb	0.565	0.6	0.53
svm_linear	0.51	0.34	0.68
svm_radial	0.54	0.41	0.67
svm_polynomial	0.49	0.05	0.93
svm_sigmoid	0.47	0.3	0.64
lg	0.55	0.46	0.64
rf	0.585	0.64	0.53
com3	0.595	0.49	0.7

**Table 11:** Summary of results for **data.bee3.3** with baseline correction.

Method	$b_{\mathrm{acc}}$	${\mathrm{acc}}_{0}$	${\mathrm{acc}}_{1}$
1nn	**0.64**	0.6	0.68
nb	**0.653**	0.76	0.547
svm_linear	**0.62**	0.52	0.72
svm_radial	0.593	0.36	0.827
svm_polynomial	0.5	0.08	0.92
svm_sigmoid	0.435	0.47	0.4
lg	**0.71**	0.66	0.76
rf	**0.615**	0.63	0.6
com3	**0.69**	0.66	0.72

**Table 12:** **Best results for THRESHOLD 0.6**: We counted the number of times the sensitivity threshold of 0.6 was exceeded. We considered variants v1 to v6. The best method was found to be svm_linear and com3 and variants v4 and v2. Slightly better were v4 and svm_linear. It is worth noting that com3 also includes svm_linear. In yellow, we highlighted the best methods and variants.

Method	*v*1	v2	v3	v4	v5	*v*6	*sum*(v1:v4)	*sum*(v1:v6)
1nn	0	3	2	4	1	2	9	12
nb	0	2	1	3	0	1	6	7
19svm_linear	4	5	5	5	6	5	19	30
svm_radial	0	3	1	2	1	0	6	7
svm_polynomial	0	0	1	1	0	0	2	2
svm_sigmoid	0	0	0	1	0	0	1	1
lg	4	3	5	3	0	0	15	15
rf	1	2	0	2	3	1	5	9
com3	4	6	4	5	−	−	19	−
sum	13	24	19	26	11	9		

**Table 13:** **Best results for THRESHOLD 0.65**: We counted the number of times the sensitivity threshold of 0.65 was exceeded. We considered variants v1 to v6. The best method was found to be svm_linear and com3 and variants v4 and v2. Slightly better were v4 and svm_linear. It is worth noting that com3 also includes svm_linear. In yellow, we highlighted the best methods and variants.

Method	v1	v2	v3	v4	v5	v6	*sum*(v1:v4)	*sum*(v1:v6)
1nn	0	1	0	1	1	0	2	3
nb	0	0	0	1	0	0	1	1
svm_linear	2	5	3	4	4	3	14	21
svm_radial	0	0	0	0	0	0	0	0
svm_polynomial	0	0	0	0	0	0	0	0
svm_sigmoid	0	0	0	1	0	0	1	1
lg	1	0	2	2	0	0	5	5
rf	1	1	0	1	1	0	3	4
com3	0	5	3	5	−	−	13	−
sum	4	12	8	15	6	3		

**Table 14:** **Best results for THRESHOLD 0.7**: We counted the number of times the sensitivity threshold of 0.7 was exceeded. We considered variants v1 to v6. The best method was found to be svm_linear and com3 and variants v4 and v2. Slightly better were v4 and svm_linear. It is worth noting that com3 also includes svm_linear. In yellow, we highlighted the best methods and variants.

Method	v1	v2	v3	v4	v5	v6	*sum*(v1:v4)	*sum*(v1:v6)
1nn	0	1	0	0	0	0	1	1
nb	0	0	0	0	0	0	0	0
svm_linear	0	3	1	2	2	1	6	9
svm_radial	0	0	0	0	0	0	0	0
svm_polynomial	0	0	0	0	0	0	0	0
svm_sigmoid	0	0	0	0	0	0	0	0
lg	0	0	0	2	0	0	2	2
rf	0	0	0	1	0	0	1	1
com3	0	1	1	1	−	−	3	−
sum	0	5	2	6	2	1		

**Table 15:** Results for the TRN-TST sample (90 percent of sites to 10 percent).

$seria_{1.2\_1}$: [1 0] -> [0.7803 0.2197]
$seria_{1.2\_10}$: [1 0] -> [0.84 0.1597]
$seria_{1.2\_13}$: [0 1] -> [0.8584 0.1415]
$seria_{2.2\_11}$: [1 0] -> [0.7495 0.2502]
$seria_{2.2\_14}$: [0 1] -> [0.8613 0.139 ]
$seria_{2.2\_17}$: [0 1] -> [0.849 0.1508]
$seria_{3.2\_1}.$: [1 0] -> [0.8296 0.1704]
$seria_{3.2\_10}$: [1 0] -> [0.8496 0.15 ]
$seria_{3.2\_11}$: [0 1] -> [0.4597 0.54 ]
$seria_{3.2\_12}$: [1 0] -> [0.8364 0.1636]
$seria_{3.2\_13}$: [0 1] -> [0.003414 0.996]
Accuracy: 72.72727489471436
